# Kimura Disease Associated with Minimal Change Disease

**DOI:** 10.4274/tjh.galenos.2020.2020.0477

**Published:** 2021-06-01

**Authors:** Rafet Eren, Enes Cömert, İlknur Mansuroğlu, Esma Evrim Doğan, Gülay Kadıoğlu

**Affiliations:** 1 University of Health Sciences Turkey, Prof. Dr. Cemil Taşcıoğlu City Hospital, Department of Hematology, İstanbul, Turkey; 2 University of Health Sciences Turkey, Prof. Dr. Cemil Taşcıoğlu City Hospital, Department of Internal Medicine, İstanbul, Turkey; 3 University of Health Sciences Turkey, Prof. Dr. Cemil Taşcıoğlu City Hospital, Department of Pathology, İstanbul, Turkey; 4 University of Health Sciences Turkey, Prof. Dr. Cemil Taşcıoğlu City Hospital, Department of Nephrology, İstanbul, Turkey

**Keywords:** Kimura disease, Lymphadenopathy, Eosinophilia, Minimal change disease

## To the Editor,

Kimura disease is a benign chronic inflammatory disease with unknown etiology, which usually presents with lymphadenopathies in the head and neck, peripheral blood eosinophilia, and elevated serum immunoglobulin E (IgE) levels. It is mostly observed in young males of Asian descent in the second and third decades of life, but sporadic cases in other ethnic groups have also been reported [1,2]. Here we present a patient with Kimura disease and concomitant nephrotic syndrome who presented with lymphadenopathies of atypical locations.

**Figure 1:**
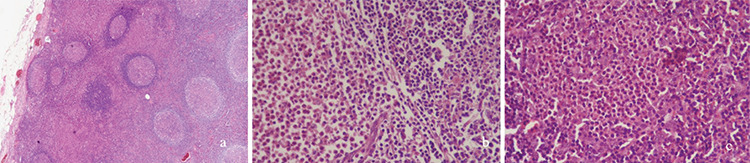
Lymph node biopsy: **(a)** markedly hyperplastic follicles with reactive germinal centers, **(b)** well-defined peripheral mantle zone, and **(c)** intense diffuse eosinophilia with formation of eosinophilic microabscesses. Paracortical plasma cells were also present. IgG4 was increased with a value of 0.8%, which was below the 40% threshold for IgG4-related disease.

A 25-year-old male patient presented with new-onset hypertension, decreased urine output, and lower extremity swelling. His past medical history was unremarkable with no history of allergies. On examination, his blood pressure was 140/100 mmHg, heart rate was 90/min, and body temperature was 37 °C. He had bilateral 2+ pitting edema of the bilateral lower extremities and an enlarged, soft, nontender 3-cm left inguinal lymph node. Laboratory evaluation results were as follows: white blood cells, 6790/µL; eosinophils, 1280/µL (18.9%); normal hemoglobin level and platelet count; serum creatinine, 0.66 mg/dL; urea, 26 mg/dL; albumin, 2.4 g/dL; triglyceride, 295 mg/dL; erythrocyte sedimentation rate, 81 mm/h; total IgE, 3318 kU/L (<87). Urinalysis showed 3+ protein and the spot urine protein/creatinine ratio was 7819 mg/g. Viral serologies and rheumatologic markers were negative. Serum C3, C4, IgG, IgA, and IgM levels were also normal. A percutaneous renal biopsy was performed for nephrotic syndrome. Pathological examination of the specimen revealed no significant changes by light microscopy and was negative for immunofluorescence, indicating minimal change disease. The patient was started on low-dose perindopril with a gradual increase to 10 mg/day. Positron emission tomography-computed tomography (CT) performed to assess any associated malignancy showed hypermetabolic activity in the inguinal and right external iliac regions (SUV_max_: 2.5). The left inguinal lymph node was excised. The pathology was reported to be consistent with Kimura disease (Figure 1). During follow-up, his creatinine levels progressively increased to 2 mg/dL and he was started on methylprednisolone at 1 mg/kg. At week 1, his creatinine regressed to baseline. At week 3, complete remission of proteinuria was achieved and the steroid was tapered slowly. Abdominal CT performed in the fourth month showed significant decrease in the number and size of lymph nodes. Steroid treatment was discontinued at the seventh month. To date, the patient has no symptoms and is being followed recurrence-free.

There are many reports of renal involvement in Kimura disease. A review of 175 patients with Kimura disease found renal involvement in 12% [3]. Renal biopsies of patients with renal involvement of Kimura disease showed mesangioproliferative glomerulonephritis, minimal change disease, focal segmental glomerulosclerosis, membranous nephropathy, membranoproliferative glomerulonephritis, and acute tubular necrosis [4]. The treatment, however, remains unclear. While excision is considered adequate in patients without renal involvement, systemic steroids are recommended in patients who have renal involvement or relapse after excision [5].

Here, we have presented a patient who was diagnosed with minimal change disease and Kimura disease and responded to steroid treatment. Kimura disease should be considered in patients investigated for lymphadenopathies in the presence of elevated serum IgE levels and renal disease.
